# Remote effects of temporal lobe epilepsy surgery: Long‐term morphological changes after surgical resection

**DOI:** 10.1002/epi4.12733

**Published:** 2023-04-25

**Authors:** T. Campbell Arnold, Lohith G. Kini, John M. Bernabei, Andrew Y. Revell, Sandhitsu R. Das, Joel M. Stein, Timothy H. Lucas, Dario J. Englot, Victoria L. Morgan, Brian Litt, Kathryn A. Davis

**Affiliations:** ^1^ Department of Bioengineering, School of Engineering & Applied Science University of Pennsylvania Philadelphia Pennsylvania USA; ^2^ Center for Neuroengineering and Therapeutics University of Pennsylvania Philadelphia Pennsylvania USA; ^3^ Department of Neuroscience, School of Engineering & Applied Science University of Pennsylvania Philadelphia Pennsylvania USA; ^4^ Department of Neurology, Perelman School of Medicine University of Pennsylvania Philadelphia Pennsylvania USA; ^5^ Department of Radiology, Perelman School of Medicine University of Pennsylvania Philadelphia Pennsylvania USA; ^6^ Department of Neurosurgery, Perelman School of Medicine University of Pennsylvania Philadelphia Pennsylvania USA; ^7^ Department of Neurological Surgery Vanderbilt University Medical Center Nashville Tennessee USA; ^8^ Department of Radiology and Radiological Sciences Vanderbilt University Medical Center Nashville Tennessee USA; ^9^ Institute of Imaging Science Vanderbilt University Medical Center Nashville Tennessee USA

**Keywords:** brain atrophy, cortical thickness, cortical thinning, neurosurgery, seizure, virtual resection

## Abstract

**Objective:**

Epilepsy surgery is an effective treatment for drug‐resistant patients. However, how different surgical approaches affect long‐term brain structure remains poorly characterized. Here, we present a semiautomated method for quantifying structural changes after epilepsy surgery and compare the remote structural effects of two approaches, anterior temporal lobectomy (ATL), and selective amygdalohippocampectomy (SAH).

**Methods:**

We studied 36 temporal lobe epilepsy patients who underwent resective surgery (ATL = 22, SAH = 14). All patients received same‐scanner MR imaging preoperatively and postoperatively (mean 2 years). To analyze postoperative structural changes, we segmented the resection zone and modified the Advanced Normalization Tools (ANTs) longitudinal cortical pipeline to account for resections. We compared global and regional annualized cortical thinning between surgical treatments.

**Results:**

Across procedures, there was significant cortical thinning in the ipsilateral insula, fusiform, pericalcarine, and several temporal lobe regions outside the resection zone as well as the contralateral hippocampus. Additionally, increased postoperative cortical thickness was seen in the supramarginal gyrus. Patients treated with ATL exhibited greater annualized cortical thinning compared with SAH cases (ATL: −0.08 ± 0.11 mm per year, SAH: −0.01 ± 0.02 mm per year, *t* = 2.99, *P* = 0.006). There were focal postoperative differences between the two treatment groups in the ipsilateral insula (*P* = 0.039, corrected). Annualized cortical thinning rates correlated with preoperative cortical thickness (*r* = 0.60, *P* < 0.001) and had weaker associations with age at surgery (*r* = −0.33, *P* = 0.051) and disease duration (*r* = −0.42, *P* = 0.058).

**Significance:**

Our evidence suggests that selective procedures are associated with less cortical thinning and that earlier surgical intervention may reduce long‐term impacts on brain structure.

**Key Points**

- Different epilepsy surgical approaches lead to distinct patterns of postoperative cortical atrophy in remote brain regions.
- Postoperative cortical thinning is greatest in the ipsilateral insula, temporal lobe, and the contralateral hippocampus.
- Patients treated with SAH have less postoperative cortical thinning than patients treated with ATL.
- The insula demonstrated the greatest focal differences in postoperative cortical thinning when comparing SAH and ATL.
- Age and preop thickness were associated with cortical thinning, indicating less structural impact from earlier surgical intervention.

## 1 INTRODUCTION

Epilepsy affects 65 million people worldwide, with one‐third of patients suffering from drug‐resistant epilepsy (DRE).^1^, ^2^ Patients with DRE bear greater risk of premature death, injury, and worsening quality of life, including psychosocial dysfunction.^3^ Surgical resection has been established as an effective treatment when a focal seizure onset zone can be localized.^4^ The temporal lobe is the most common localization site, with temporal lobe epilepsy (TLE) accounting for approximately half of DRE cases.^1^, ^5^, ^6^, ^7^ Anterior temporal lobectomy is the most common surgical approach for patients with TLE.^8^, ^9^ However, in recent years surgical options have expanded to include more selective resections,^10^ focal laser ablations,^11^ and intracranial neural stimulation.^12^ These less invasive surgical approaches seek to achieve seizure control while preserving cognitive function and reducing surgical comorbidities.^13^, ^14^ However, comparisons of cognitive outcomes and seizure freedom between traditional and selective procedures have been mixed, which likely reflects a complex trade‐off between preserving functional brain tissue and removing epileptogenic regions.^15^, ^16^, ^17^, ^18^

Across institutions, numerous surgical procedures are performed for epilepsy management, including anterior temporal lobectomy (ATL), selective amygdalohippocampectomy (SAH), lesionectomy, and focal ablation. Substantial evidence, including a rigorous randomized controlled trial and multi‐institution meta‐analyses, indicates that ATL is superior to continued antiepileptic medication in up to 70% of cases.^4^, ^19^ However, evidence indicates that more focal surgery, such as laser ablations, lead to better cognitive outcomes.^11^, ^18^ Despite a growing list of surgical options, clinicians lack quantitative methods for assessing the impact of different procedures on brain structure and function. Although the cause of accelerate brain atrophy in epilepsy is still an active area of debate,^20^ recent evidence suggests that ongoing seizures are a contributing factor.^21^, ^22^ While preoperative cortical thinning is relatively well studied, comparatively little is known about how surgery affects long‐term brain morphometry. Studies have demonstrated that some patients who are initially seizure‐free after surgery eventually relapse,^23^ and while the exact cause of these relapses is not known, alteration in brain structure has shown predictive value for determining which patients will relapse.^24^ Quantitative neuroimaging could provide additional insight into differences in long‐term seizure freedom and cognitive outcomes between surgical treatments, thus providing clinical guidance and improving patient outcomes.^25^

Surgical resection has remote consequences outside the removed tissues, such as white matter atrophy;^26^ however, these remote changes are not well understood. While many studies demonstrated ongoing seizures lead to cortical atrophy,^27^ few quantify changes seen between presurgical and postsurgical imaging.^28^, ^29^ Characterizing downstream effects of surgery is important as changes in brain structure may negatively affect patient outcome, leading to decreased rates of seizure freedom or increased cognitive side‐effects. Furthermore, given a planned resection, quantitative measurements in combination with brain connectivity analyses could be used to model remote effects of a proposed brain surgery.^30^, ^31^ There remains a great need for quantitative methods to guide clinical decisions about therapy.

In this study, we use advanced computational imaging tools to quantify changes throughout the brain and hypothesize that there are measurable changes in cortical thickness caused by epilepsy surgery. We propose that these changes are a function of the surgical technique employed. To understand the effects of surgical approach on brain structures, we developed quantitative techniques to evaluate postoperative imaging for cortical thickness changes in adjacent and remote brain regions. We quantify downstream effects of two common surgical procedures, ATL and SAH, in 36 patients who underwent TLE surgery. We developed a semiautomated method for segmenting resection cavities and considering the resection extent when computing cortical thinning in the brain. We publicly share our code for assessing postoperative changes in cortical thickness to accelerate translation into clinical practice.

## 2 METHODS

### 2.1 Patients

We recruited 37 patients who underwent surgery for localization‐related epilepsy across two institutions, Hospital of the University of Pennsylvania (HUP, N = 14) and Vanderbilt University Medical Center (VUMC, N = 22). The protocol was approved by the Vanderbilt University Institutional Review Board and the Institutional Review Board of the University of Pennsylvania. All participants gave informed consent. Patients had similar age distributions across institutions (HUP: 39.4 ± 13.0 years, VUMC: 36.5 ± 12.8 years) and were treated with either ATL (N = 22) or SAH (N = 14). All SAH patients were treated with transcortical approach through the middle temporal gyrus.^32^ Clinical variables collected include gender, age at surgery, age at onset, disease duration, seizure lateralization, interscan interval, and surgical outcomes. For patients treated at HUP, age at onset, disease duration, and surgical outcomes were not available, and all related statistics only include VUMC patients. Surgical outcomes were assessed using the Engel classification system and binarized into Engel I (more favorable outcome) and Engel II‐IV (less favorable outcome) groups. Patient demographics and clinical characteristics are listed in Table 1 and Table S1.

**TABLE 1:** Demographics of patients included in study.

Procedure	ATL	SAH	Total
Number of participants	22	14	36
Gender (Female/Male)	14/8	8/6	22/14
Age at surgery (Years, mean ± SD)	39.6 ± 12.6	34.5 ± 13.0	37.6 ± 13.0
Age at onset (Years, mean ± SD)	25.6 ± 10.1	15.8 ± 11.5	19.4 ± 12
Duration (Years, mean ± SD)	17.4 ± 13.8	23.6 ± 16.1	21.4 ± 15.6
Seizure side (Left/Right)	7/15	5/9	12/24
Interscan interval (Years, mean ± SD)	1.68 ± 1.27*	2.95 ± 1.23*	2.17 ± 1.40
1 Year outcome (Engel I/Engel II–IV)	5/3	10/4	15/7
Latest outcome (Engel I/Engel II–IV)	5/3	9/5	14/8

*Note*: An asterisk denotes a significant difference between groups. Some clinical variables, including age at onset, disease duration, and surgical outcome, are only available for patients treated at VUMC (N = 22).

Abbreviations: ATL, anterior temporal lobectomy; SAH, selective amygdalohippocampectomy; SD, standard deviation.

### 2.2 Image acquisition

All patients underwent a clinical epilepsy neuroimaging protocol both preoperatively and postoperatively (on average 2 years after surgery) as part of their standard care. The post‐resection imaging protocol was acquired on average 26 months (range: 5 months to 5 years) after implant and resection. For each patient, 1 mm isotropic pre‐implant T1‐weighted MRI (T1w) and post‐resection T1w images were acquired. Preoperative and postoperative images were collected on the same scanner to avoid the confounds of inter‐scanner variability in cortical thickness estimation. Patients at HUP were imaged using a Siemens Trio scanner with a 64‐channel head coil and received a T1w with the following acquisition parameters (1 mm isotropic, TE = 3.87 ms, TR = 1.62 s, flip‐angle = 15) with an average interscan interval of 11 months (range: 5 months to 2 years 8 months). Patients at VUMC were imaged using a Phillips 3 T scanner with a 32‐channel head coil and received a T1w acquired with the following acquisition parameters (1 mm isotropic, TE = 4.61 ms, TR = 8.9 ms, flip‐angle = 8) with an average interscan interval of 3 years (range: 1 year 1 month to 5 years).

### 2.3 Selection of surgical approach

At VUMC, patients are typically chosen for SAH when there is a high level of confidence that the seizures are mesial (e.g., seemingly mesial semiology, MTS on MRI, hippocampal onset assessed on intracranial implant). ATL is typically performed when clinicians are relatively confident that the seizures are localized to the unilateral temporal lobe, but there is less evidence and confidence that they are mesial (e.g., atypical semiology, nonlesional MRI, and possible lateral involvement assessed on intracranial implant). All HUP patients included in this study were treated with ATL.

### 2.4 Resection segmentation

Patient images were analyzed using Advanced Normalization Tools (ANTs).^33^, ^34^ Preoperative images were registered to the postoperative image, and the Atropos algorithm^35^ was used to segment images into six tissue types: cerebral spinal fluid (CSF), gray matter (GM), white matter (WM), deep gray matter (DGM), cerebellum, and brainstem. In postoperative images, the resection area is predominantly labeled as CSF, while the same voxels would be labeled as GM or WM in preoperative images.

To generate an initial, automated estimation of the resection zone, we subtracted postoperative and preoperative CSF probability maps. Prior to subtraction, a gaussian smoothing kernel (sigma = 2) was passed over the probability maps to avoid large contrast due to misalignment.^36^ Voxels with greater than 25% increases in CSF probability from preoperative to postoperative timepoints were considered as candidates for the resection mask. We constrained the segmentations to only include voxels original classified as GM or WM in preoperative images.^37^ To eliminate stray voxels, only the largest contiguous cluster of voxels in the resection mask was retained. All resection zones estimates were manually reviewed and corrected by a neuroradiologist (JMS, 8 years of experience) using ITK‐SNAP.^38^

To compare the extent of resection associated with each procedure, probability maps were generated for each combination of surgical technique (ATL or SAH) and hemisphere (left or right). For each subject, preoperative and postoperative T1w images were rigidly coregistered and resection masks were transformed into preoperative space. Preoperative imaging was then registered to the MNI ICBM152 template^39^ and the transformation was applied to the resection mask, warping it into MNI space. A probability map with values ranging from 0% to 100% was generated by averaging resection masks in MNI space across all patients within each procedure and hemispheric combination.

### 2.5 Cortical thinning estimation

We assessed cortical thickness using an adaptation of the well‐validated ANTs longitudinal cortical thickness pipeline.^33^, ^34^, ^40^, ^41^, ^42^, ^43^ For patients with cortical resections, the standard ANTs cortical thickness pipeline causes significant image distortion during deformable registration of postoperative and template images.^44^, ^45^ This distortion results from white matter and gray matter in the postoperative image erroneously being pulled into the resection cavity to better match the template image intensity values (Figure S1), and ultimately leads to downstream inaccuracies in cortical thickness estimation.

Recently, a new deep learning enhanced version of ANTs was released (ANTsPyNet), which eliminates the need for deformable registration during cortical thickness estimation.^34^ We further modified the longitudinal cortical thickness pipeline from this package to account for the postoperative resection zone during single‐subject template (SST) generation. When generating an SST, voxel intensity values are reduced in the resection zone because preoperative and postoperative images are averaged. We used the resection segmentation to impute preoperative values into the affected region of the SST. This generates a template image that closely aligns with the patients imaging, without reduced intensity values in the resection zone. Examples of tissue segmentation near the resection sites are illustrated in Figure S2.

Mean cortical thickness was estimated for preoperative and postoperative images across a set of 62 regions in the Desikan‐Killiany‐Tourville (DKT) atlas.^46^, ^47^ The ipsilateral amygdala, hippocampus, entorhinal cortex, and parahippocampal regions were excluded from our analysis, as these regions are completely or nearly completely removed during resective surgery. Cortical thinning was calculated as the difference of mean postoperative and preoperative cortical thickness. To account for differences in follow‐up time between subjects, we divided by the interscan interval to yield annualized cortical thinning rates. A visual description of the analytical pipeline is provided in Figure S3. Patterns of cortical thinning were visualized using the BrainNet Viewer toolbox.^48^

### 2.6 Statistical analysis

The two treatment groups, ATL and SAH, were compared for differences in age at surgery, gender, interscan interval, mesial temporal sclerosis status, and lateralization using two‐sample *t*‐tests or chi‐squared tests. Global differences in cortical thinning across all regions outside the resection zone were compared between groups using a two‐sample *t*‐test. Differences in cortical thinning were assessed across 62 individual brain regions from the DKT atlas. Brain regions were assessed as either ipsilateral or contralateral to the resection site. Across brain region comparisons, false‐discovery rate (FDR) correction was applied to account for multiple comparisons. Pearson's correlation and *t*‐tests were used to related cortical thinning to clinical variables.

### 2.7 Preoperative cortical thinning

**FIGURE 1:**
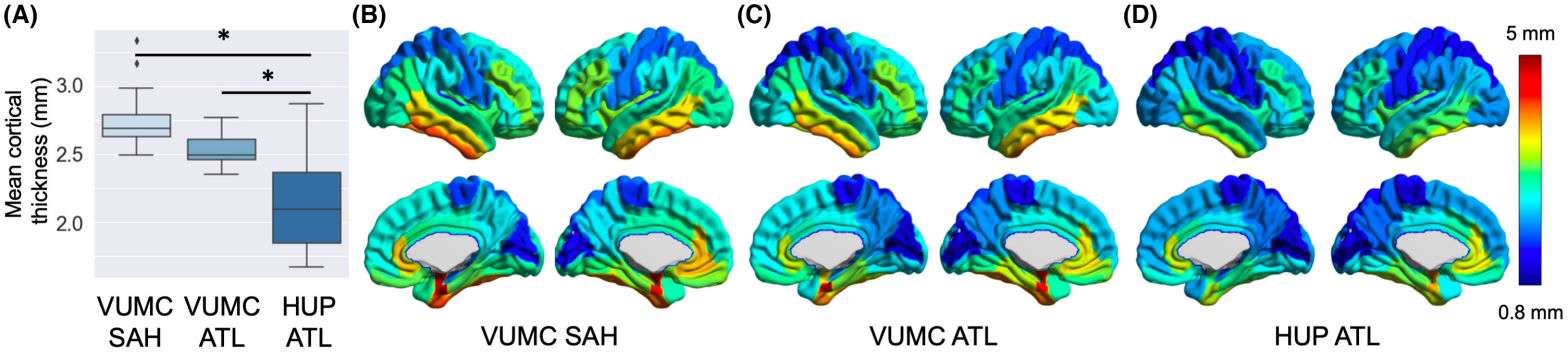
Preoperative cortical thickness. Patients had similar preoperative cortical thickness values regardless of which surgical procedure they eventually received. However, there was an effect across sites, such that patients treated at HUP had lower preoperative cortical thickness values. (A) Patients treated at HUP with ATL had significantly lower preoperative cortical thickness compared with SAH (two‐sample *t* test: *t* = 4.64, *P* < 0.001) and ATL (two‐sample *t* test: *t* = 3.50, *P* = 0.002) patient treated at VUMC. (B‐D) Regional preoperative cortical thickness values can be seen for patients treated at (B) VUMC with SAH, (C) VUMC with ATL, and (D) HUP with ATL. Cortical thickness patterns are similar for both VUMC groups, while patients treated at HUP with ATL have markedly lower cortical thickness values globally. ATL, anterior temporal lobectomy; HUP, Hospital of the University of Pennsylvania; SAH, selective amygdalohippocampectomy; VUMC, Vanderbilt University Medical Center.

To assess for selection bias between scan sites and surgical treatment groups, we compared mean preoperative cortical thickness for patients treated with SAH at VUMC, ATL at VUMC, and ATL at HUP. For VUMC patients, those treated with ATL trended toward lower preoperative cortical thickness, though ATL and SAH treatment groups were not significantly different in preoperative cortical thickness between (two‐sample *t*‐test: *t* = 2.08, *P* = 0.051), indicating similar preoperative states (Figure 1A). However, HUP patients treated with ATL had significantly lower mean cortical thickness compared with VUMC patients treated with SAH (two‐sample *t*‐test: *t* = 4.64, *P* < 0.001) and ATL (two‐sample *t*‐test: *t* = 3.50, *P* = 0.002). Regional patterns of cortical thickness appear similar between all groups, although with a global decrease in thickness across all regions in HUP patients (Figure 1B‐D). Given that there was no significant difference in age between any combination of scan sites or treatment groups, it is possible the lower cortical thickness values at HUP may be attributable to scanner differences between the sites. Additionally, cortical thinning is expected to be greater in patients with longer interscan intervals, which could bias analyses toward greater cortical thinning in the SAH treatment group. To address the discrepancy in interscan interval and potential scanner‐site confounds, all subsequent analyses will compare annualized cortical thinning rates.

### 2.8 Data and code availability

All code related to resection segmentation, cortical thickness estimation, and statistical analysis are available at: https://github.com/tcama/remote_effects. Data supporting the findings of this study are available from the corresponding author upon reasonable request.

## 3 RESULTS

### 3.1 Demographics

Thirty‐seven patients were included in this study (14 patients from the Hospital of the University of Pennsylvania and 22 patients from the Vanderbilt University Medical Center). One patient was excluded due to motion degradation in postoperative imaging. Table 1 summarizes important clinical demographics for the study patients. For patients treated at HUP, age at onset, disease duration, and surgical outcome were not available. There was no difference in mesial temporal sclerosis status between centers (*χ* ^2^ = 0.15, *P* = 0.93). There was no statistically significant difference between patients treated with SAH or ATL in patient age at surgery (two‐sample *t*‐test: *t* = 1.15, *P* = 0.26), gender (*χ* ^2^ = 0.15, *P* = 0.92), or side of seizure onset (*χ* ^2^ = 0.06, *P* = 0.97). There was a significant difference in the interscan interval, or time between preoperative and postoperative imaging (two‐sample *t*‐test: *t* = 2.87, *P* = 0.007), such that patients treated with ATL had a shorter interval (1.68 ± 1.27 years) than those treated with SAH (2.95 ± 1.23 years).

### 3.2 Resection extent

**FIGURE 2:**
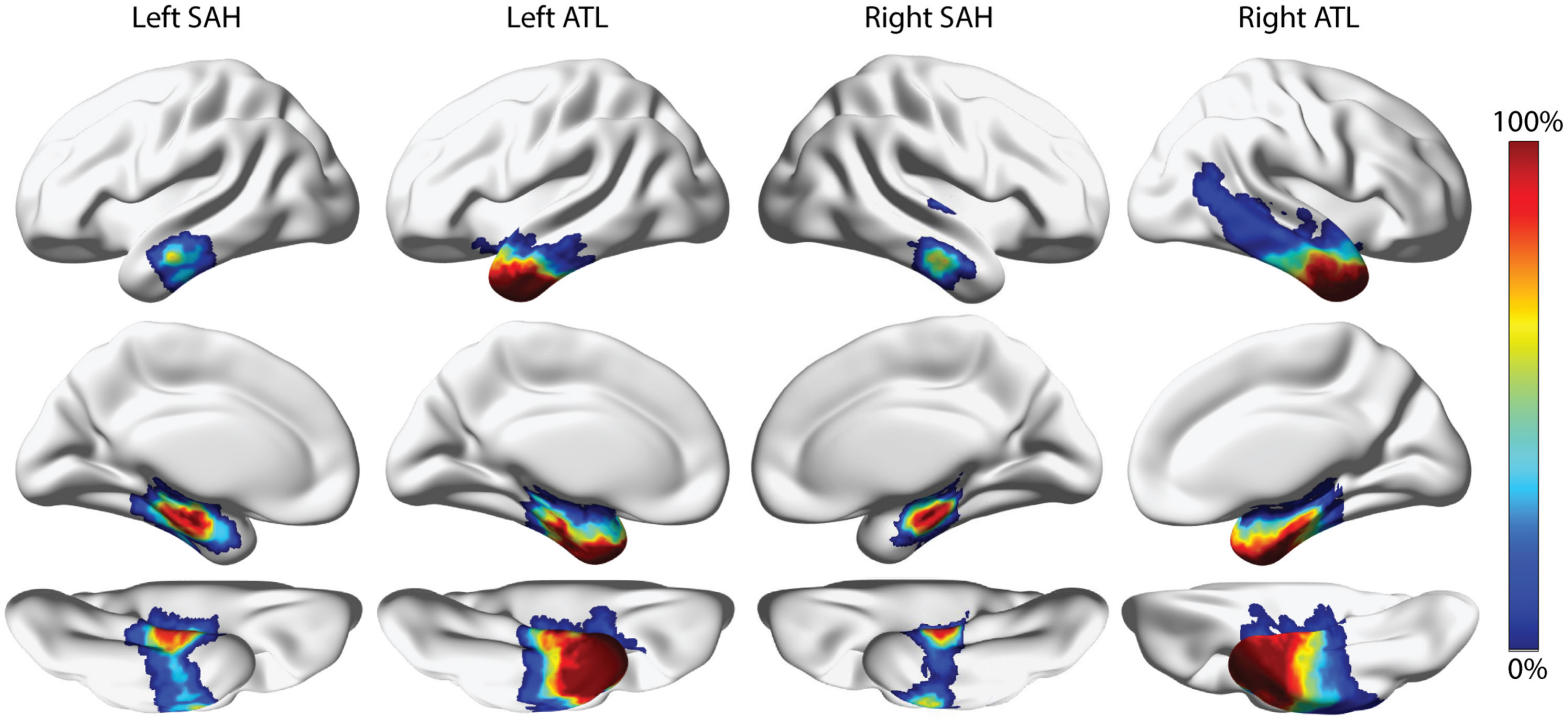
Probability maps for each surgical group. For each surgical technique (SAH or ATL) and hemisphere combination (left or right), a probability map was generated to visualize the extent of the resection procedures. Each subject's T1w imaging was registered to the MNI ICBM152 template and the manually drawn resection masks were warped to MNI space. A probability map with values ranging from 0% to 100% was generated by averaging the resection masks in MNI space. Warmer colors indicate areas with high surgical resection overlap between patients. In both hemispheres, patients treated with ATL have the highest overlap in the anterior temporal pole, with probability of resection reducing as you travel posteriorly. Patients treated with SAH have the largest overlap in the mesial temporal lobe and have reduced resection coverage compared with ATL patients.

To illustrate the extent of resections, we generated probability maps for each combination of surgical technique and hemispheric lateralization, as seen in Figure 2. Surgical margins are based on the patient's language lateralization, with wider margins for epilepsy surgery in the non‐dominant hemisphere.^49^ The left hemisphere is typically the language‐dominant hemisphere, which results in larger resection margins being associated with right hemisphere surgeries in our dataset. In both hemispheres, patients treated with SAH have reduced resection margins compared with patients treated with ATL.

### 3.3 Cortical thinning

**FIGURE 3:**
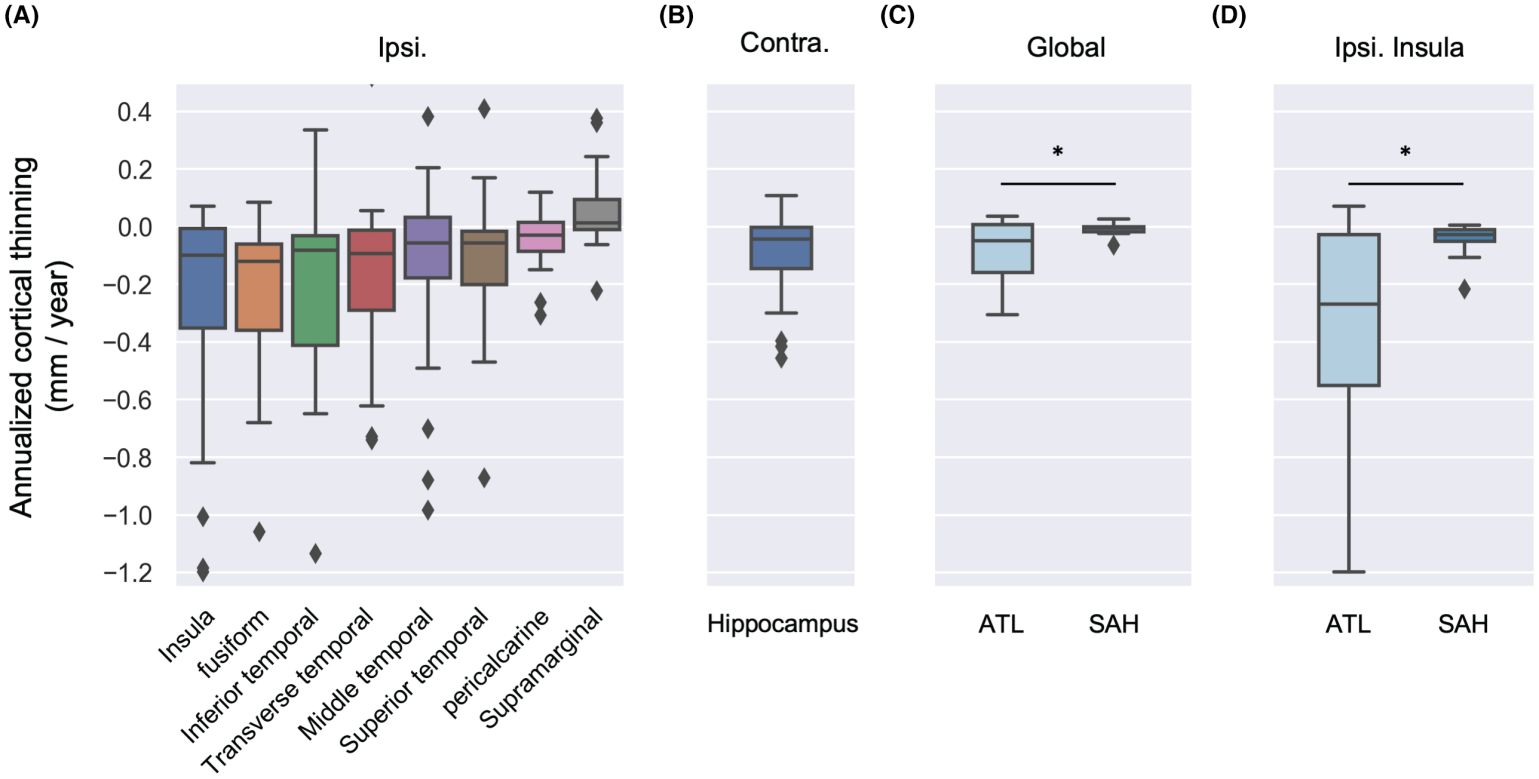
Changes in postoperative cortical thickness. This figure illustrates significant changes in cortical thickness. (A) Ipsilateral cortical thinning across both procedures was seen in the insula, the fusiform cortex, temporal lobe regions, and the pericalcarine. A cortical thickness increase was seen in the supramarginal gyrus. (B) In the contralateral hemisphere, cortical thinning was seen in the hippocampus. (C) Globally, there was significantly greater cortical thinning in patients treated with ATL, (D) with a focal difference in the insula.

In the hemisphere ipsilateral to surgical resection, across both procedures significant postsurgical cortical thinning was seen in several temporal lobe regions adjacent to the surgical site (Figure 3A), including the inferior temporal gyrus (*P* = 0.012, FDR corrected), middle temporal gyrus (*P* = 0.040, FDR corrected), superior temporal gyrus (*P* = 0.035, FDR corrected), and transverse temporal gyrus (*P* = 0.040, FDR corrected). Ipsilateral cortical thinning was also seen in the fusiform cortex (*P* < 0.001, FDR corrected), pericalcarine (*P* = 0.040, FDR corrected), and insula (*P* = 0.003, FDR corrected). Interestingly, there was also a significant cortical thickness increase in the ipsilateral supramarginal gyrus (*P* = 0.040, FDR corrected). In the contralateral hemisphere, cortical thinning was observed in the hippocampus (*P* = 0.017, Figure 3B).

To assess whether there was a global difference in cortical thinning between patients treated with ATL and SAH, annualized cortical thinning was compared between groups using a two‐sample *t*‐test (Figure 3C). Globally, there was significantly more cortical thinning in patients treated with ATL over those treated with SAH (two‐sample *t*‐test: *t* = 2.99, *P* = 0.006). Patients treated with ATL had elevated annualized cortical thinning (0.08 ± 0.11 mm per year), while those treated with SAH had much lower rates of cortical thinning (0.01 ± 0.02 mm per year). There was no global difference in cortical thinning based on left or right hemispheric lateralization.

**FIGURE 4:**
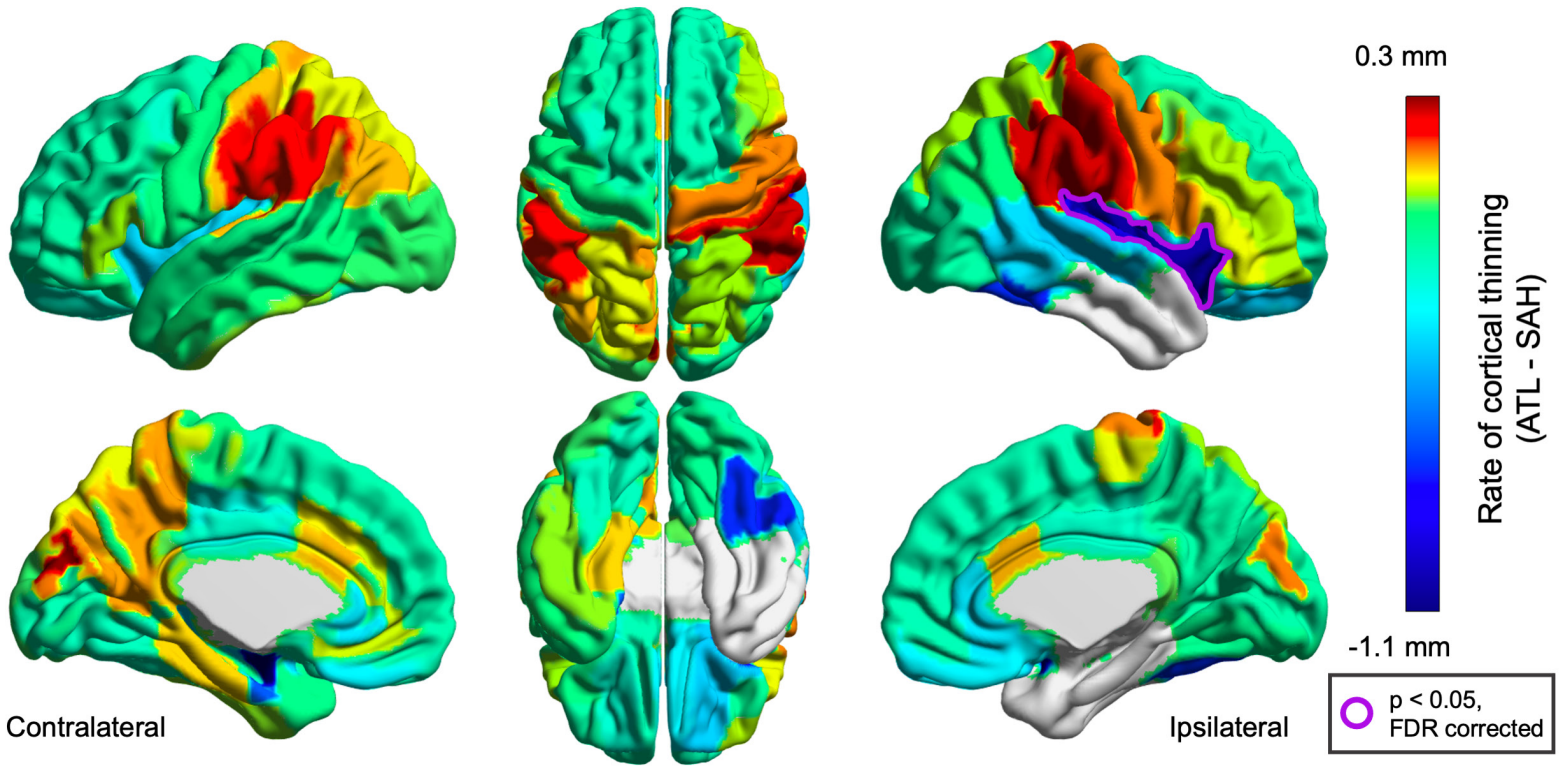
Differences in cortical thinning rates between ATL and SAH. This figure compares cortical thinning rates between patients treated with ATL and SAH at the level of individual brain regions. Areas with cool colors indicate greater cortical thinning in patients treated with ATL, while warmer colors indicate areas with greater cortical thinning in SAH patients. Regions with significant differences after FDR correction are outlined in purple. Globally, rates of cortical thinning are higher for patients treated with ATL, with the largest differences in the ipsilateral insula, ipsilateral temporal lobe outside the resection zone, and contralateral hippocampus. The supramarginal gyrus is a notable exception, where cortical thickness increases more in patients treated with ATL.

At the individual brain region level, there was significantly more cortical thinning in the ipsilateral insula for patients treated with ATL (*P* = 0.039, Figure 3D). There were no differences between ATL and SAH in the contralateral hemisphere. Although statistical differences in many regions did not persist after FDR correction, overall the extent of cortical thinning was greater in patients treated with ATL compared with SAH, as visualized in Figure 4, with the notable exception of the supramarginal gyrus which showed a greater degree of cortical thickness increase.

### 3.4 Relating cortical thinning to clinical variables

**FIGURE 5:**
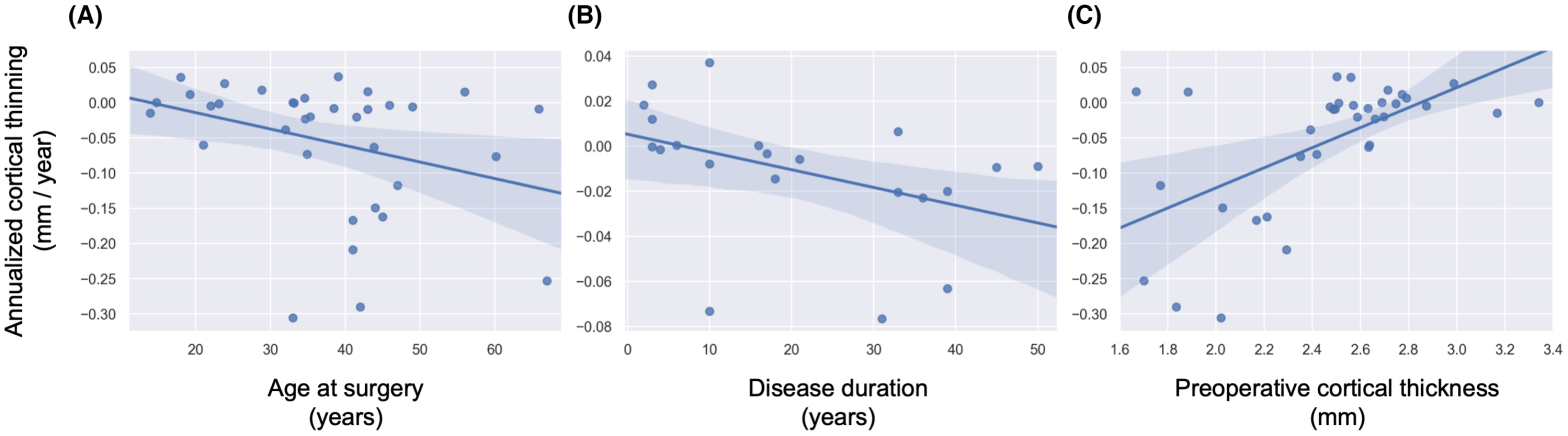
Correlations between clinical variables and cortical thinning. This figure illustrates correlations between cortical thinning and a patient's (A) age at surgery (*r* = −0.33, *P* = 0.051), (B) disease duration (*r* = −0.42, *P* = 0.058), and (C) their preoperative cortical thickness (*r* = 0.60, *P* < 0.001). The disease duration analysis includes a subset of 22 patients with available data; all other correlations examine the full dataset.

To better understand the factors that contribute to accelerated cortical thinning, we correlated individual thinning rates with clinical variables of the patients. We analyzed their age at surgery, age at onset, disease duration, mean preoperative cortical thickness, and Engel outcome. Analyses of age at onset, disease duration, and Engel outcome were limited to a subset of 22 patients with available data. We found that an individual's annualized cortical thinning rate was associated with their preoperative cortical thickness (*r* = 0.60, *P* < 0.001) and to a lesser extent though not significantly with their age at surgery (*r* = −0.33, *P* = 0.051) and disease duration (*r* = −0.42, *P* = 0.058; Figure 5). This result means patients that were older, patients with a longer history of epilepsy, and patients with lower preoperative cortical thickness experienced greater rates of cortical thinning. Such age‐related variables are difficult to distinguish as a patient's age at surgery also relates to both their disease duration (*r* = 0.64, *P* = 0.002) and their preoperative cortical thickness (*r* = −66, *P* < 0.001). There was no relationship with age at onset (*r* = 0.20, *P* = 0.39). Finally, we compared cortical thinning rates between patients that had more favorable outcomes (Engel I) and less favorable outcomes (Engel II‐IV). There was no significant difference in cortical thinning between surgical outcomes groups, for either their outcome measured 1 year after surgery (*P* = 0.27) or their most recent Engel outcome available (*P* = 0.23).

## 4 DISCUSSION

Epilepsy is associated with brain atrophy, particularly hippocampal sclerosis in temporal lobe epilepsy patients.^50^, ^51^ It is known that cortical atrophy in epilepsy is not confined to the epileptogenic zone but rather permeates throughout the neocortex.^27^, ^52^, ^53^, ^54^, ^55^ While ongoing seizures and cortical atrophy are relatively well studied, comparatively little is known about how epilepsy surgery subsequently affect cortical thinning.^28^, ^29^ The lack of focus on postsurgical treatment groups is partly due to the technical difficulties of working with postoperative imaging. In this study, we conducted a whole‐brain comparative analysis of longitudinal cortical thinning between two epilepsy surgical techniques, anterior temporal lobectomy, and selective amygdalohippocampectomy. We developed and publicly released semi‐automated software for assessing longitudinal cortical thinning while accounting for resected tissue.

Our most important finding was that patients treated with SAH had less postoperative cortical thinning than ATL patients. There was a global effect, with further analysis revealing that the largest driver was the ipsilateral insula. Across both procedures, we found cortical thinning in the ipsilateral fusiform cortex, insula, pericalcarine, and temporal lobe regions outside the resection zone. Cortical thinning was also seen in the contralateral hippocampus. Interestingly, the supramarginal gyrus showed a postoperative cortical thickness increase. We found that age‐related variables, including age at surgery, disease duration, and preoperative cortical thickness, were predictive of postoperative cortical thinning rates.

We speculate that cortical thinning patterns and regional postoperative differences based on surgical approach are reflective of underlying network connectivity between affected cortices and downstream regions.^56^ Temporal, limbic, and insular brain regions form an integrated functional network and prior studies have observed that TLE cortical thinning patterns overlap the hippocampal structural connectivity network.^21^, ^57^ Epilepsy accelerates cortical atrophy with regions connected to the epileptogenic zone most likely to be afflicted.^21^, ^58^ The overlap between cortical thinning patterns and structural connectivity maps of the putative epileptogenic zone indicate potential for using cortical thinning to localize the epileptic focus or differentiate epilepsy subtypes.^59^

Our study adds to a growing body of work that validates cortical thickness measurements as an important biomarker for epilepsy. In a recent longitudinal case–control study, Galovic et al. observed that epilepsy patients had a twofold increase in the rate of annualized cortical thinning compared with healthy controls (TLE 0.024 ± 0.061 mm per year vs healthy controls 0.011 ± 0.029 mm per year).^21^ In our work, we observed that ATL patients had higher rates of postoperative annualized cortical thinning 0.08 ± 0.11 mm per year, while SAH patients showed minimal progressive cortical thinning (0.01 ± 0.02 mm per year) similar to cortical thinning rates reported in healthy controls. The accelerated cortical thinning and differences between the surgical approaches may result from greater Wallerian degeneration and loss of functional connections in ATL patients, though tractography studies would be necessary to confirm this hypothesis. All SAH patients in our study were treated with a transcortical approach through the middle temporal gyrus; however, different SAH approaches may yield different patterns of atrophy.

Another recent study compared preoperative and postoperative rates of annualized cortical thinning and demonstrated that epilepsy surgery restored cortical thinning rates to the same baseline as healthy controls.^22^ The authors posit that epilepsy surgery has long‐term neuroprotective effects and promote earlier surgical intervention. In our study, we found that greater postoperative cortical thinning was associated with older patients and to some extent a longer disease duration. Our evidence similarly suggests that early surgical intervention minimizes negative long‐term impacts on brain structure.

Several studies have compared selective and traditional surgical approaches, with most citing similar seizure outcomes and superior cognitive outcomes for selective procedures.^13^, ^14^ However, the cause of these differences in cognitive outcomes remains poorly understood. Selective procedures may preserve more functional gray matter near the focal lesion, or traditional resections may cause greater neurologic injury through trauma to white matter tracts or vasculature. In the present study, we demonstrated differences in downstream cortical thinning between SAH and ATL. Interestingly, in mesial temporal lobe epilepsy patients Bettus et al. observed a decrease in ipsilateral temporal lobe connectivity while conversely connectivity increased in the contralateral hemisphere.^60^ They proposed this may reflect a compensatory mechanism to preserve normal cognitive function. In our study, we observed a cortical thickness increase in the ipsilateral supramarginal gyrus which was more pronounce in ATL patients. This could represent a similar compensatory response in somatosensory association cortex. Furthermore, recent work has demonstrated long‐term atrophy and functional connectivity changes in the contralateral hippocampus postoperatively.^61^, ^62^ In our data, we saw cortical thinning in the contralateral hippocampus, which corroborates work by Elliott et al. demonstrating contralateral hippocampal volume loss after surgery. These findings could be a structural analog for functional connectivity observations. However, postoperative neuropsychiatric outcomes and functional or structural connectivity studies would be necessary to better understand this association.

Our quantitative cortical thickness method could provide clinical utility during surgical planning. Network‐based models provide an in silico approach to mapping seizure onset zones and resection zone targeting. Such methods allow clinicians to map the role of each node using network‐control methodology,^30^, ^63^ with network nodes representing ECoG or sEEG electrodes. However, these methods focus solely on the resected tissue and do not account for downstream network effects. Brain regions identified in our work could be used to modify network‐based models to account for downstream effects. Additionally, epilepsy surgery is associated with changes to structural connectivity in remote brain regions.^64^ By modeling resections on structural connectivity and relating them to postoperative cortical thinning patterns, we can better understand how focal treatments affect brain structure globally.

Three major study limitations are a small cohort size, lack of clinical variables, and heterogeneity in surgical approach and interscan interval. These factors often limit studies of drug‐resistant epilepsy as obtaining consistent longitudinal data is difficult and patients are lost to follow‐up. Our study may be underpowered to detect subtle differences in cortical thinning, especially in subjects with shorter interscan intervals. Additionally, we analyzed regional differences as ipsilateral or contralateral to the surgery, which may introduce variability and precludes assessing hemispheric differences. Finally, there was a difference in preoperative cortical thickness between patient groups, which could contribute to differences in cortical thinning rates. While the study may be underpowered, we applied FDR correction to minimize potential Type I errors and annualized cortical thinning analyses were used to address interscan interval heterogeneity and differences in baseline cortical thickness. Additionally, procedural choice is not random, but rather is informed by characteristics of an individual's disorder (e.g., presence of mesial temporal sclerosis). This presents a potential confound, making it difficult to determine whether effects are caused by the surgical approach or the individual's characteristics that lead them to receive that procedure. This study represents an initial foray to uncover possible regions of interest that are likely affected by resection; however, larger studies should be conducted to establish definitive differences between treatment groups. Studies should also control for potential confounds, such as age at surgery, preoperative cortical thickness, and disease duration, which we found to correlate with cortical thinning rates. Additionally, a comparison to age‐matched controls would be desirable to establish whether surgical patients return to similar cortical thinning rates as healthy controls. Finally, our study lacks behavioral outcome measures (e.g., memory and language). Future studies should include neuropsychiatric outcome variables and additional surgical approaches.

## DISCLOSURES

Neither of the authors has any conflict of interest to disclose.

## ETHICAL PUBLICATION STATEMENT

We confirm that we have read the Journal's position on issues involved in ethical publication and affirm that this report is consistent with those guidelines.

## Supporting information


Appendix S1
