# Supplementary material for: Remote effects of temporal lobe epilepsy surgery: Long‐term morphological changes after surgical resection
Source: Epilepsia Open. 2023 Apr 25;8(2):559–70. doi: 10.1002/epi4.12733 (PMC10235552; doi:10.1002/epi4.12733)
Supplement: Supplementary file 1 — Appendix S1 [file EPI4-8-559-s001.docx]

**SUPPLEMENTAL TABLES & FIGURES**

| **Table S1 –Demographics of patients at each study site** | | | | | | | |
| --- | --- | --- | --- | --- | --- | --- | --- |
|  |  | HUP |  | VUMC |  | Total |  |
| Number of participants |  | 14 |  | 22 |  | 36 |  |
| Gender (Female / Male) |  | 10/4 |  | 12/10 |  | 22/14 |  |
| Age at surgery (Years, mean ± SD) |  | 39.4 ± 13.0 |  | 36.5 ± 12.8 |  | 37.6 ± 13.0 |  |
| Age at onset (Years, mean ± SD) |  | ND |  | 19.4 ± 12.0 |  | 19.4 ± 12.0 |  |
| Duration (Years, mean ± SD) |  | ND |  | 21.4 ± 15.6 |  | 21.4 ± 15.6 |  |
| Seizure side (Left / Right) |  | 7/7 |  | 5/17 |  | 12/24 |  |
| Surgical approach (ATL / SAH) |  | 14/0 * |  | 8/14 * |  | 22/14 |  |
| Interscan interval (Years, mean ± SD) |  | 0.9 ± 0.6 * |  | 3.0 ± 1.2 * |  | 2.2 ± 1.4 |  |
| 1 year outcome (Engel I / Engel II-IV) |  | ND |  | 13/9 |  | 13/9 |  |
| Latest outcome (Engel I / Engel II-IV) |  | ND |  | 12/10 |  | 12/10 |  |

Notes: An asterisk denotes a significant difference between groups. Some clinical variables, including age at onset, disease duration, and surgical outcome are only available for patients treated at VUMC (N=22). Abbreviations: HUP, Hospital of the University of Pennsylvania; VUMC, Vanderbilt University Medical Center; SD, standard deviation; ND, no data; MTS, mesial temporal sclerosis; ATL, anterior temporal lobectomy; SAH, selective amygdalohippocampectomy.


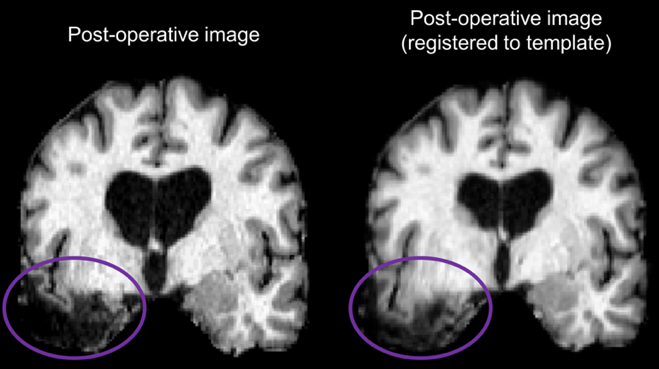


**Figure S1.** **Tissue deformation errors**. The original image (left) and the image after registration to a template (right) are shown with purple circles highlighting the area of greatest tissue deformation caused by the registration process.


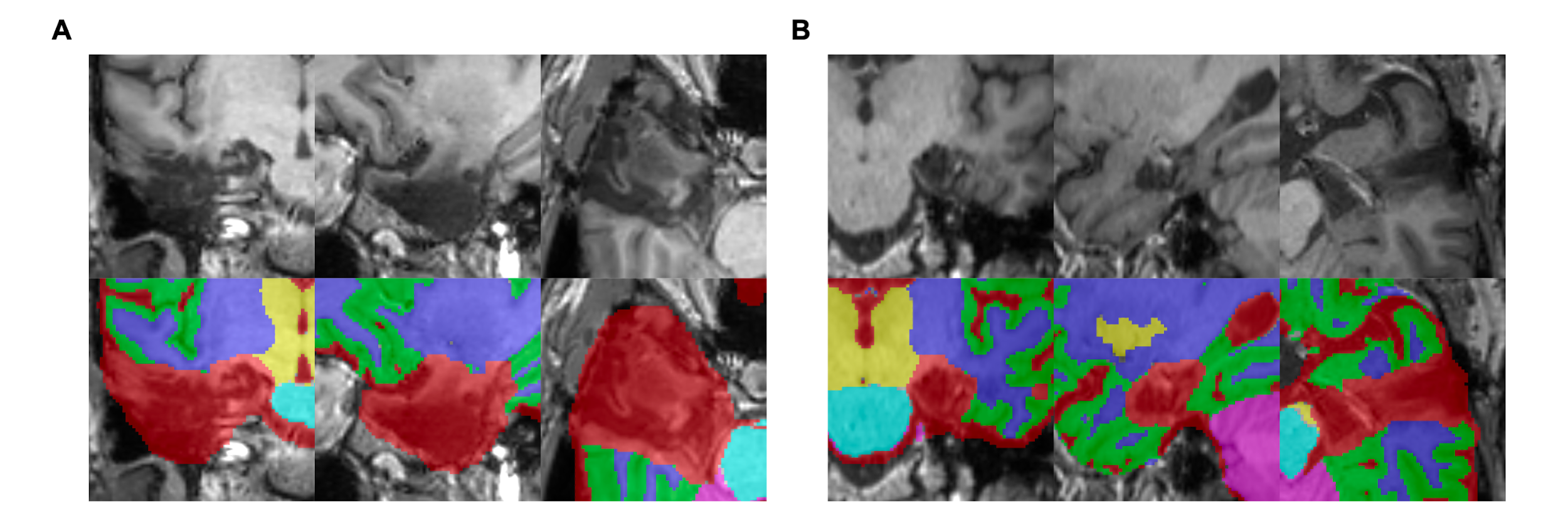


**Figure S2. Tissue segmentation near surgical site**. This figure illustrates Atropos tissue segmentations near the surgical sites a patient treated with (A) ATL and (B) SAH. The top row is the T1-weighted MRI, the bottom row is the T1-weighted MRI with the tissue segmentation overlaid. Tissue types included in the segmentations are gray matter (green), white matter (blue), cerebrospinal fluid (red), deep gray matter (yellow), brainstem (light blue), cerebellum (pink).


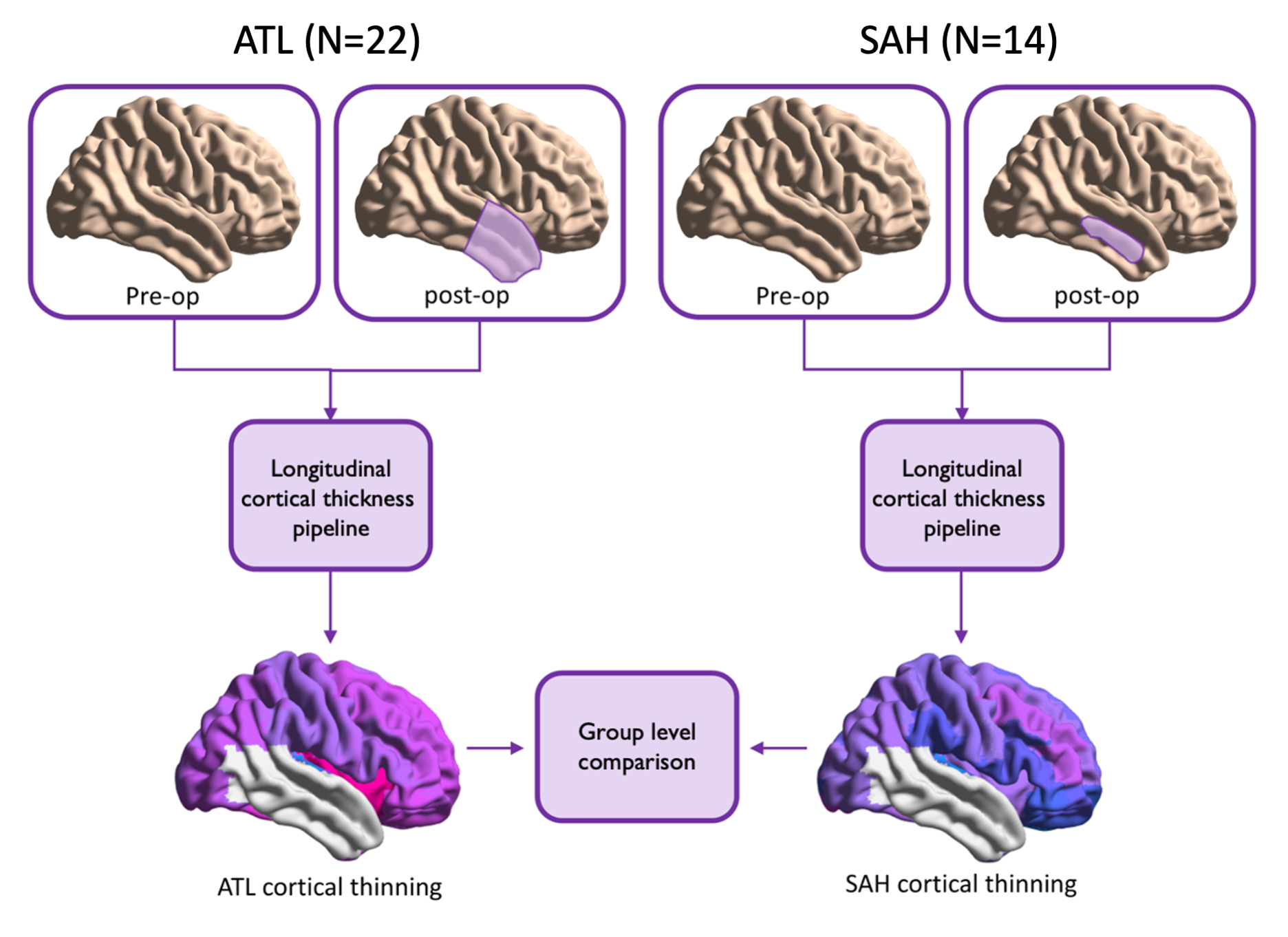


**Figure S3.** **Analytical pipeline**. Pre- and post-operative imaging for 22 patients treated with ATL and 14 patients treated with SAH were processed through a modified version of the ANTs cortical thickness pipeline. Subsequently each brain region is compared in group level statistical tests. Abbreviations: ATL, anterior temporal lobectomy; SAH, selective amygdalohippocampectomy.
